# Dietary lysophospholipids enhance broiler performance, immune response, meat quality, and mitigate oxidative stress

**DOI:** 10.3389/fimmu.2025.1572314

**Published:** 2025-08-29

**Authors:** Yahya Eid, Sabria A. El-Soud, Mostafa Z. Gamel, Seham El-Kassas, Mahmoud M. Azzam, Alessandro Di Cerbo, Ahmed A. Elolimy, Mahmoud Alagawany, Abeer A. Kirrella

**Affiliations:** ^1^ Department of Poultry Production, Faculty of Agriculture, Kafrelsheikh University, Kafrelsheikh, Egypt; ^2^ Animal, Poultry and Fish Breeding and Production, Department of Animal Wealth Development, Faculty of Veterinary Medicine, Kafrelsheikh University, Kafrelsheikh, Egypt; ^3^ Department of Animal Production, College of Food and Agricultural Sciences, King Saud University, Riyadh, Saudi Arabia; ^4^ School of Biosciences and Veterinary Medicine, University of Camerino, Matelica, Italy; ^5^ Department of Integrative Agriculture, College of Agriculture and Veterinary Medicine, United Arab Emirates University, Al Ain, United Arab Emirates; ^6^ Poultry Department, Faculty of Agriculture, Zagazig University, Zagazig, Egypt

**Keywords:** broilers, oxidative stress, dexamethasone, lysophospholipids emulsifiers, intestinal health

## Abstract

**Background:**

The current study evaluated the impact of lysophospholipid emulsifiers’ (LPLs) dietary incorporation on ameliorating the negative impacts of oxidative stress in broilers.

**Methods:**

A total of 270 2-week-old male Avian 48 chicks were randomly divided into six experimental groups. The first group fed a basal diet (BD) only, while the second group (+DEX) received BD containing 2 mg/kg dexamethasone. The third and fourth groups consisted of birds fed a BD containing 0.5 and 1 g of LPLSs/kg, respectively. The fifth and sixth groups, received BD containing 1 mg/kg dexamethasone and were supplemented with 0.5 and 1 g of LPLs, respectively.

**Results:**

Separate supplementation of LPLs significantly improved the broilers’ growth as confirmed by increasing final weight, body gain, and FI with improved feed conversion ratio (FCR) (*P* < 0.05). LPLs also improved the carcass yield (carcass, breast, and thigh muscle percentages, *P* = 0.0001) and meat quality (water-holding capacity, *P* < 0.05; tenderness, *P* < 0.05; pH, *P* < 0.001; and color, *P* < 0.05), with notable improvement in intestinal and liver histology and significantly increased intestinal villi length and width (*P* < 0.001). Furthermore, LPLs improved the serum levels of globulin (*P* < 0.01), creatinine (*P* < 0.001), LDL cholesterol (*P* < 0.001), HDL cholesterol (*P* < 0.01), and triglycerides (*P* < 0.001). Immune and antioxidant levels, as well as LPLs’ dietary supplementation, distinctly increased the phagocytic activity and index, total antioxidants, superoxide dismutase, catalase, and glutathione peroxidase, with a marked reduction in malondialdehyde (MDA) (*P* < 0.05). However, feeding dexamethasone negatively impacted the birds’ performance, confirmed by a marked retardation of the birds’ growth as manifested by lowering final body weight, gain, and increasing FCR, along with poor carcass yield and increased abdominal fat accumulation (*P* < 0.05). The dexamethasone-associated negative impacts were ameliorated with the combined LPL dietary supplementation.

**Conclusion:**

Dietary supplementation of LPLs at 0.5g level could effectively mitigate the adverse effects of oxidative stress in broilers, improving the growth performance, immune response, intestinal health, and meat quality of broiler chickens under normal and stressful conditions.

## Introduction

The worldwide expansion of the poultry industry is primarily driven by the continuously increasing demand for high-quality protein sources (1–3), although poultry, particularly broiler chickens, faces many challenges, such as oxidative stress and metabolic disorders, that adversely impair growth performance, immunity, and overall health (4, 5). These challenges are exacerbated by intensive poultry farming and the high metabolic and growth rates of broilers, underscoring the need for innovative nutritional strategies to effectively address these challenges (6).

Oxidative stress, a critical challenge in broiler production, results from an imbalance between the production of reactive oxygen species (ROS) and the body’s antioxidant defenses (8, 9). This imbalance negatively affects growth performance, compromises immune function, and reduces meat quality, leading to substantial economic losses (7). The broilers’ rapid growth rates and environmental stressors further exacerbate the negative impacts of oxidative stress, leading to reduced water-holding capacity, loss of color stability, and diminished nutritional value in meat (10–13). These alterations not only reduce consumer appeal but also shorten the shelf-life and marketability of poultry products.

Recent research has highlighted the potential effectiveness of dietary emulsifiers in enhancing poultry health and productivity (14, 15). Exogenous emulsifiers have gained attention as feed additives that enhance nutrient digestibility and overall digestive efficiency in broilers (16), especially during their early life stages, as they absorb fewer fats due to their immature digestive systems and the lower secretion of lipases and bile salts (17, 18). This insufficiency in the production of mixed micelles in the broilers’ small intestine results in reduced fat absorption and digestion (19, 20). Emulsifiers play a critical role in enhancing lipid digestion by compensating for the immature bile salt secretion system, thereby improving lipid absorption (21, 22). Enhanced lipid digestion and utilization ensure better fat utilization, providing an essential energy source for rapid growth (23), particularly during the critical early growth stages. By improving lipid digestion and reducing energy expenditure, emulsifiers enhance growth performance and feed conversion ratios (18, 24–27). Among the various types of emulsifiers, lysolecithin has shown a considerable potential for strengthening fat utilization and nutrient digestibility. It enhances lipid metabolism and ensures greater energy availability for growth and development. Additionally, lysolecithin positively impacts gut health by maintaining mucosal integrity and supporting the growth of beneficial microbial populations. These effects together enhance the performance, improve the feed efficiency, and promote the overall health status of poultry (28–30).

Additionally, dietary emulsifiers have been shown to have benefits in mitigating the negative impact of stress in the poultry industry (31). Despite aforementioned literatures which demonstrated the promising contribution of exogenous dietary emulsifiers to develop nutritional strategies that enhance broiler performance, improve product quality, and maintain animal welfare, most of these studies focused on the effect of LPLs on the low-energy diet and how emulsifier supplementation could compensate this shortage particularly during the early stage of growth (starter phase) when the digestive system is underdeveloped, with limited bile salt and lipase secretion, which reduces fat digestion efficiency. Additionally, there is a dearth of studies examining the effect of dietary supplementation on LPLs in response to various stressors, such as nutritional glucocorticoid-associated stress, particularly during the growing phase when the bird’s energy requirements are increasing. So, the current study aimed to explore how dietary supplementation with lysophospholipid emulsifiers could improve the nutritive quality of regular standard diet to improve the broilers’ growth, antioxidants, meat quality, and non-specific immune response, either normal or under the glucocorticoids’ oxidative stress during the growth phase of life (from 15 to 35 days old).

## Materials and methods

### Ethical approval

This work was approved by the Ethics Committee of Local Experimental Animals Care at Kafrelsheikh University (KFS-IACUC/235/2024) and was conducted according to the guidelines of Kafrelsheikh University, Egypt.

### Birds’ management and experimental design

In this experiment, a total of 270 1-day-old male (Avian 48) chicks were obtained from a commercial hatchery of El-Sabeil Poultry Company, ElGharbeia, Egypt, with an average body weight of 50.31 ± 0.27 g. The chicks were individually weighed and randomly allocated to six equal groups with five replicates of nine birds in each. The birds were kept for the first 2 weeks without any supplementation and received only the standard soybean–corn basal diet (BD) (**Table 1**) to minimize the influence of early-life stressors, such as transportation and mortality, and to provide a more stable number for each group. Moreover, it is also to ensure a relatively mature digestive and metabolic system, which is effective in evaluating the impact of different nutritional supplementation (32, 33). Therefore, at 14 days old, the birds received dexamethasone at 2 mg/kg diet (34) and lysophospholipid emulsifiers (LPLs) at 0.5 and 1 g/kg diet based on the findings of El-Sayed (35). Solbi et al. (36) reported an enhancement of the broilers’ growth performance and gut health following lysophospholipid supplementation, including lecithin at 0.5 and 1.00 g/kg diet. Based on this, the experimental groups included the first group (negative control, -DEX) and fed basal diet (BD) only (average weight was 547.4 ± 6.92 g), while the second group (positive control, +DEX) of birds received BD containing 2 mg/kg dexamethasone (average weight was 543.98 ± 2.92 g). The third (0.5 g LPLs) and fourth (1 g LPLs) groups were birds fed BD containing 0.5 and 1 g of LPLs/kg diet, respectively (average body weight: 549.56 ± 3.49 and 543.49 ± 3.94 g, respectively), whereas the fifth (0.5 LPLs + DEX) and sixth (1 LPLs + DEX) groups were birds that received BD containing 2 mg/kg dexamethasone and supplemented with 0.5 and 1 g of LPLs/kg diet, respectively (average body weight: 542.40 ± 4.04 and 539.27 ± 5.01 g, respectively). The differences in initial weights were statistically analyzed using one-way ANOVA, which revealed no statistically significant differences.

**Table 1:** Composition and calculated chemical analysis of the basal experimental diet.

Ingredient (g/kg)	Starter (1–17 – days)	Grower (18–35 days)
Yellow corn	510	549
Soybean meal, 46%	365	315.8
Corn gluten meal, 60%	38	47
Soya oil	36.25	40.35
Calcium carbonate	15	14.8
Dicalcium phosphate	19.5	18
Salt	2.5	2.5
Sodium sulfate	1.8	1.6
DL-Methionine, 99%	2.6	2.1
l-Lysine HCl, 98%	2.8	2.6
l-Threonine	1.2	0.9
Choline chloride, 60%	0.8	0.8
Premix^a^	3	3
Anticoccidia	0.2	0.2
Anticlostridia	0.1	0.1
Antimycotoxin biology	0.25	0.25
Silica	1	1
Chemical analysis on DM basis		
AME (kcal/kg)	3,000	3,100
Crude protein, %	23.0	21.5
Fat, %	6.3	6.9
LYS, %	1.28	1.15
M and C, %	0.95	0.87
THR, %	0.86	0.77
Calcium, %	0.96	0.87
Available P, %	0.48	0.44
Sodium, %	0.16	0.16
Chloride, %	0.23	0.23

a Premix (Hero mix^®^ (Hero Pharm, Cairo, Egypt)) composed of (per 3 kg): vitamin A: 12,000,000 IU; vitamin D3: 2,500,000 IU; vitamin E: 10,000 mg; vitamin K3: 2,000 mg; vitamin B1: 1,000 mg; vitamin B2: 5,000 mg; vitamin B6: 1,500 mg; vitamin B12: 10 mg; niacin: 30,000 mg; biotin: 50 mg; folic acid: 1,000 mg; pantothenic acid: 10,000 mg; manganese: 60,000 mg; zinc: 50,000 mg; iron: 30,000 mg; copper: 4,000 mg; iodine: 300 mg; selenium: 100 mg; and cobalt: 100 mg.

The birds were fed a standard broiler diet (**Table 1**), which was formulated based on the nutritional requirements of Avian 48 Cobb broilers (37) and to meet the nutritional requirements according to NRC guidelines. The composition and ingredients of the three-phase diet (starter, 0–10 days; grower, 11–24 days; and finisher, 25–35 days) are presented in **Table 1**. The feeding program consisted of three phases: starter (from 0 to 12 days), growing (13 to 24 days), and finisher from 25 to 35 days. The birds had free access (*ad libitum*) to feed and water throughout the experimental period.

Moreover, chicks were housed in floor pens (1 m × 1.5 m) with a room temperature that started at 33 ± 1°C on the first day and was gradually reduced by 3°C every week until it reached 23 ± 1°C by the end of the experiment (35 days). The relative humidity (RH%) was maintained between 50% and 70% throughout the experimental period. The birds received 23 L:1 D from 0 to 7 days; after that, they received 18 L:6 D for the remaining days.

The lysophospholipid (LPL) emulsifier LYSOFORTE^®^ was purchased from Kemin Europa NV, Herentals, Belgium. The primary active ingredient in LYSOFORTE is lysolecithin, which is produced through an enzymatic process in which soy lecithin is converted into LPLs. At the same time, the synthetic steroid dexamethasone (Dexazone^®^, 0.5 mg dexamethasone/tablet) was purchased from El-Kahira Pharmaceuticals and Chemical Industry Company (Shoubra, Cairo Governorate, Egypt).

### Experimental procedures and samplings

#### Growth performance evaluation

Chicken body weight (BW) and feed consumption were recorded weekly to the nearest grams and used to calculate the average body weight gain (BWG) and feed conversion ratio (FCR). FCR was calculated based on the following formula: FCR = feed consumed (g)/weight gain (g).

#### Samples collection

At the end of the experiment (at 35 days), after 6 h of fasting, 15 birds per treatment (three birds from each replicate) were randomly selected, weighed, and used for sample collection. From each bird, two blood samples were drawn from the wing vein using two types of sterile syringes (heparinized and non-heparinized). Non-heparinized blood was used for serum separation, which was stored at -20°C for further assessment of biochemical parameters. Serum samples were obtained from the non-heparinized blood by centrifugation at 2,500 *g* and 4°C for 15 min (38). Heparinized blood was used to evaluate the phagocytic activity and index (39). After blood collection, the birds were slaughtered, kept for complete bleeding, and then eviscerated. After that, the carcass weight, abdominal fat (the fat pad surrounding the gizzard and abdominal cavity), liver, heart, spleen, breast, and thigh muscle were collected and individually weighed. Then, two liver specimens were collected from each bird; one specimen was used for histopathological investigation and fixed in 10% formalin. The second liver specimens were stored at -20°C for further investigation. Jejunum samples were also collected and fixed in 10% formalin and used for intestinal histomorphological evaluation.

#### Serum biochemical parameter

Total protein, albumin, globulin, total cholesterol, high and low-density lipoproteins (HDL and LDL), alanine aminotransferase (ALT), aspartate aminotransferase (AST), triglycerides, and creatinine were colorimetrically measured using specific commercial kits (Diamond Diagnostics, Cairo, Egypt) according to the procedure outlined by the manufacturer.

#### Antioxidant activity

The activities of some antioxidant enzymes, including glutathione peroxidase (GPx), catalase (CAT), and superoxide dismutase (SOD), along with total antioxidant capacity (TAC) and malondialdehyde (MDA) concentration, were estimated in liver samples (15 samples/treatment) (40). In this regard, the liver samples were homogenized in sterile cold potassium phosphate buffer (pH 7) by an electric homogenizer, and then the obtained liver homogenates were centrifuged at 4,020 *g* for 15 min at 4°C, and the supernatant was used for the assessment of the antioxidant enzyme activities using a UV–VIS spectrophotometer. Accordingly, the activity of SOD in liver homogenate (U/g tissue) was determined using a commercial colorimetric kit from Bio-Diagnostic (Giza, Egypt), and the absorbance was measured at 560 nm over 5 min. Calibration was done using a SOD standard, and the intra- and inter-assay coefficients of variability (CV%) were <10%. CAT activity (U/g tissue) was determined using a commercial kit obtained from Bio-diagnostic (Giza, Egypt) (41). The principle of CAT determination is based on its reaction with a known quantity of H_2_O_2_ that stops after 1 min using a CAT inhibitor. In the presence of peroxidase, the remaining hydrogen peroxide reacts with 3,5-dichloro-2-hydroxybenzene sulfonic acid and 4-aminophenazone to form a chromophore with a color intensity inversely proportional to the amount of CAT in the sample. The absorbance was measured at 510 nm over 3 min, and the samples were standardized against blank samples and H_2_O_2_ standard (500 mM/L). The intra- and inter-assay coefficients of variability (CV%) were <10%. The GPx activity (U/g of tissue) was also evaluated using a commercial kit (Bio-diagnostic, Giza, Egypt) at 340 nm, with *R*² >0.95 and CV% <10%. MDA concentration (nmol/g of tissue) was measured at 534 nm using commercial kits from Bio-diagnostic, Giza, Egypt, with <10% reported for the intra- and inter-assay coefficients of variability. For total antioxidant capacity (TAC), it was also measured using a commercial kit supplied from Bio-diagnostic, Giza, Egypt, based on the removal of exogenously supplied hydrogen peroxide (H_2_O_2_) in the samples by its content of antioxidants, where a certain amount of the given substrate H_2_O_2_ is removed with the antioxidants in the samples, and the residues of H_2_O_2_ are colorimetrically measured at 505 nm. Briefly, the H_2_O_2_ substrate was first diluted 1,000 times, then about 0.5 mL was added to a blank, and the tested samples were mixed well and incubated for 10 min at 37°C. After that, the working reagent (chromogen and the enzyme buffer) was added to the blank and tested samples, mixed, and incubated for 5 min at 37°C. The absorbances of both blank (A_B_) and tested samples (A_SA_) were immediately determined against water at 505 nm. The TAC was calculated in millimole per liter (mmol/L) using the equation: TAC = (A_B_ - A_SA_) × 3.33. This assay had <10% for the intra- and inter-assay coefficients of variability.

#### Phagocytosis assay

The phagocytic functional assay was performed *in vitro* using *Candida albicans* (39) to evaluate the influence of LPL dietary supplementation on the phagocytic activities of immune cells and nonspecific immunity. Briefly, equal volumes of 100 μL of fresh blood, *C. albicans* suspension (equivalent to 1 × 106), and fetal bovine serum (South American) were mixed and incubated at 37°C for 30 min. Then, the mixture was centrifuged at 1,500 rpm for 10 min, and 5 µL of the resuspended cells was used for blood smear preparation. After that, the blood smears were stained using rapid field stain (polychrome methylene blue and eosin), and the slides were examined under a light microscope (Leica DM500; Leica Microsystems, Japan). The phagocytic activity (PA) and phagocytic index (PI) were calculated for 15 birds from each group (three birds/replicate). The PA equals the percentage of phagocytic cells that engulfed yeast cells, whereas PI equals the total number of phagocytized yeast cells divided by the number of phagocytic cells. The CV% of the inter- and intra-assay was less than 10%.

#### Histomorphometry parameters

The jejunum and liver specimens were fixed in 10% neutral buffered formalin. After 24 h, the samples were transferred to 70% alcohol, then dehydrated in an ascending series of ethanol, cleared in xylene, and impregnated and embedded in paraffin wax. Sections of 5 µm were cut using a Leica rotatory microtome (RM 20352035; Leica Microsystems, Wetzlar, Germany) and mounted on glass slides. The prepared tissue sections were deparaffinized in xylene and rehydrated in a descending series of ethanol, until reaching distilled water, before undergoing conventional staining with hematoxylin and eosin (H&E) (42, 43). The stained sections were examined under a light microscope (Leica DM500; Leica Microsystems, Japan). Morphometric analysis was conducted using an automated image analysis system (ImageJ; Bethesda, MD, USA) to measure villus height, villus width, crypt depth, and muscularis thickness (44).

#### Determination of pH, color, tenderness, and water-holding capacity

At the end of the experiment, a total of 15 samples of breast muscle/treatments was randomly selected for the determination of quality characterization of chicken meat. The pH value was determined using an electrical pH meter (Bye model 6020, USA) (45). The intensity of color brightness of the meat extract was also determined (46). Briefly, 10 g of meat was shaken with 22.5 mL distilled water in a darkened space for 10 min. Then, the intensity of the filtrate’s color (absorbance) was determined using a spectrophotometer at 542 nm (47). Meat tenderness and water-holding capacity were also measured (48). Briefly, approximately 0.3 g of minced meat was placed under Whatman No. 41 ashless filter paper and pressed with a 1-kg weight for 10 min. As a result of pushing, two zones were developed on the filter paper. After that, the surface areas of these zones were measured using a planimeter. Tenderness (in cm^2^) was determined by the surface area of the internal zone caused by the meat pressing. The water-holding capacity was represented by the difference between the area of the inner zone and the area of the outer zone (water-holding capacity = area of outer zone – area of inner zone).

### Statistical analysis

To determine significant differences among the various treatments, the results were statistically analyzed using one-way ANOVA followed by Tukey’s *post-hoc* tests in IBM SPSS 22. During the analysis, a *post-hoc* power analysis and partial eta squared (*η*²) were performed using the observed data. The analysis revealed that the *post-hoc* power of all assessed parameters was >0.80 with larger *η*² >0.14 at *α* = 0.05, indicating that the sample size used in this study was reasonably sufficient to detect biologically relevant differences and would strengthen the validity and reproducibility of the findings. Results were considered statistically significant at *P <*0.05 and presented as means ± SE. GraphPad Prism 9 (^©^GraphPrism Software, La Jolla, CA, USA) was used for graphical presentation and figure preparation.

## Results

### Growth performance and relative organ weights

**Figure 1:**
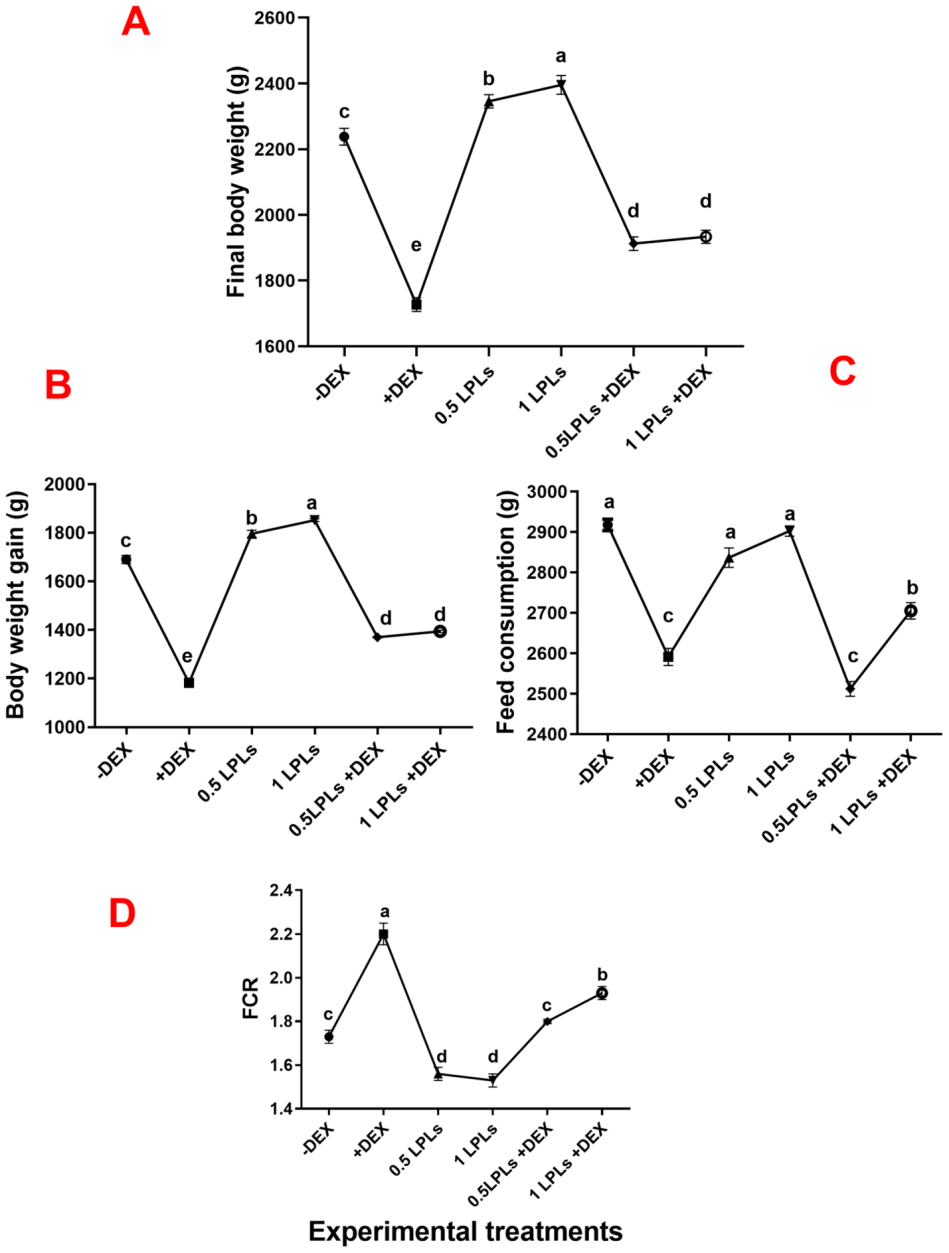
Effect of lysophospholipid dietary emusifiers on the growth performance of Avian 48 broilers. -DEX denotes [negative control and fed basal diet (BD) only], +DEX (positive control, birds received BD containing 2 mg/kg dexamethasone); 0.5 LPLs and 1 LPLs refer to birds fed BD containing 0.5 and 1 g of LPLSs/kg diet, respectively, whereas the DEX + 0.5 LPLs and DEX + 1 LPLs represent birds receiving BD containing 2 mg/kg dexamethasone and supplemented with 0.5 and 1 g of LPLs/kg, respectively. The results are expressed as mean ± SE. Different letters denote statistical significance at *P <*0.05. *Post-hoc* power = 1.00 for final body weight, body gain, FI, and FCR, and partial eta squared (*η*²) = 0.997, 0.996, 0.969, and 0.962, respectively. Part **(A–D)** show Final body weight (g), Body weight gain (g), Feed consumption (g), and FCR, respectively.

**Figure 1** displays the influence of the separate and concurrent dietary presence of various levels of LPLs and dexamethasone on the growth performance of Avian 48 broilers. Compared to the non-supplemented birds (negative control, -DEX), the separate LPL dietary supplementation (0.5 and 1 LPLs), particularly at 1 g/kg diet, significantly enhanced the broilers’ growth performance, as evidenced by increased final body weight and gain and improved FCR (*P* < 0.001). On the other hand, the dietary presence of dexamethasone suppressed the growth performance as confirmed by reduced final body weight and gain and increased FCR compared to the control group (*P* < 0.001). Interestingly, the concurrent presence of LPLs with DEX in the broilers’ diet alleviated the adverse reduced growth performance caused by dexamethasone exposure which was confirmed by restored body weight and gain (*P* < 0.001). Despite the fact that they were higher than that of the dexamethasone-treated group (+DEX), they were still less than the control (-DEX) and LPL-treated groups (*P* < 0.001). For feed intake, dexamethasone significantly reduced the feed intake (*P* < 0.001), though the two supplemented doses of LPLs (0.5 and 1 g) did not alter the amount of feed consumption compared to the control group (*P* = 0.0845 and 0.490, respectively). Moreover, the combined LPL supplementation to the DEX-exposed groups significantly restored the birds’ ability to eat as evidenced by an increase in the amount of feed consumed (*P* < 0.001). Therefore, according to the FI and body gain results, the FCR was distinctly increased in the DEX-exposed group (*P* < 0.001), while the LPL-supplemented groups displayed improved values for FCR (*P* < 0.05 and *P* < 0.01 for 0.5 and 1 g LPLs, respectively). Additionally, including LPLs in the diet with DEX significantly improved the FCR values (*P* < 0.001 and *P* < 0.01).

The influence of LPL supplementation on the relative organ weights of broiler birds undergoing oxidative stress using dexamethasone (+DEX) is shown in **Table 2**. The dietary supplementation of LPLs at 0.5 and 1 g/kg diet significantly increased the carcass yield and the thigh and breast muscle weights compared to the control group (-DEX) and other groups (*P* < 0.001 and *P* < 0.01). However, dexamethasone dietary exposure (+DEX) prominently reduced the carcass, thigh muscle, and breast muscle weights (*P* < 0.001). The weights of the carcass, thigh, and breast muscles compared to the positive control (+DEX) were measured with a concurrent dietary supplementation of 0.5 and 1 g of LPLs with DEX (*P* < 0.001). The abdominal fat weight was significantly increased in the DEX-exposed group (+DEX) compared to the control non-supplemented group (*P* < 0.01), which was reduced in a dose-dependent manner when LPLs were combined with DEX in the diet (most significantly reduced at 1 g LPLs) (*P* < 0.001).

**Table 2:** Effects of dietary supplementation of lysophospholipids (LPLs) on relative organ weights of broiler chickens subjected to oxidative stress.

Parameter g/100 g body weight	Negative control (-DEX)	Positive control (+ DEX)	0.5 LPL_S_	1 LPL_S_	0.5 LPLs +DEX	1 LPLs +DEX	*p*-value	*Post-hoc* power	*η*²
Carcass	65.33 ± 0.39b	53.54 ± 0.75d	75.59 ± 1.22a	73.00 ± 1.08a	59.31 ± 2.34c	56.35 ± 1.3cd	<0.001	1.00	0.918
Breast muscle	23.57 ± 0.59b	17.30 ± 1.11d	26.18 ± 0.58a	25.47 ± 0.77ab	19.74 ± 0.84c	17.77 ± 0.39cd	<0.001	1.00	0.896
Thigh muscle	15.82 ± 0.28a	12.57 ± 1.0c	15.36 ± 0.37a	15.62 ± 0.40a	14.62 ± 0.55ab	12.94 ± 0.75bc	<0.001	1.00	0.885
Liver	1.7475 ± 0.11c	2.82 ± 0.26a	1.68 ± 0.07c	2.52 ± 0.23c	1.76 ± 0.07b	2.30 ± 0.32ab	<0.01	0.879	0.669
Spleen	0.078 ± 0.02	0.060 ± 0.004	0.075 ± 0.02	0.092 ± 0.019	0.060 ± 0.001	0.063 ± 0.002	0.635	0.189	0.239
Abdominal fat	1.16 ± 0.06c	1.79 ± 0.09a	0.632 ± 0.04cd	0.73 ± 0.06d	1.53 ± 0.21b	2.29 ± 0.09b	<0.001	0.999	0.827
Heart	0.55 ± 0.013c	0.72 ± 0.051a	0.55 ± 0.010c	0.59 ± 0.041c	0.61 ± 0.033ab	1.47 ± 0.041ab	<0.05	0.962	0.735

Values are expressed as means ± standard error. Mean values with different letters in the same row differ significantly at *P <*0.05.

DEX, dexamethasone; *η*², partial eta squared.

Moreover, compared to the non-supplemented group, DEX exposure significantly increased the liver and heart weights (*P* < 0.01 and 0.042, respectively), which were intermediately restored to normal weight when LPLs were combined with DEX (*P* < 0.05), whereas LPLs alone at 0.5 and 1 g supplementation levels did not alter the liver and heart weights compared to the control. For spleen weight, there were no significant differences in weight among the different groups.

### Influences of LPLs and DEX on serum biochemical parameters

**Figure 2:**
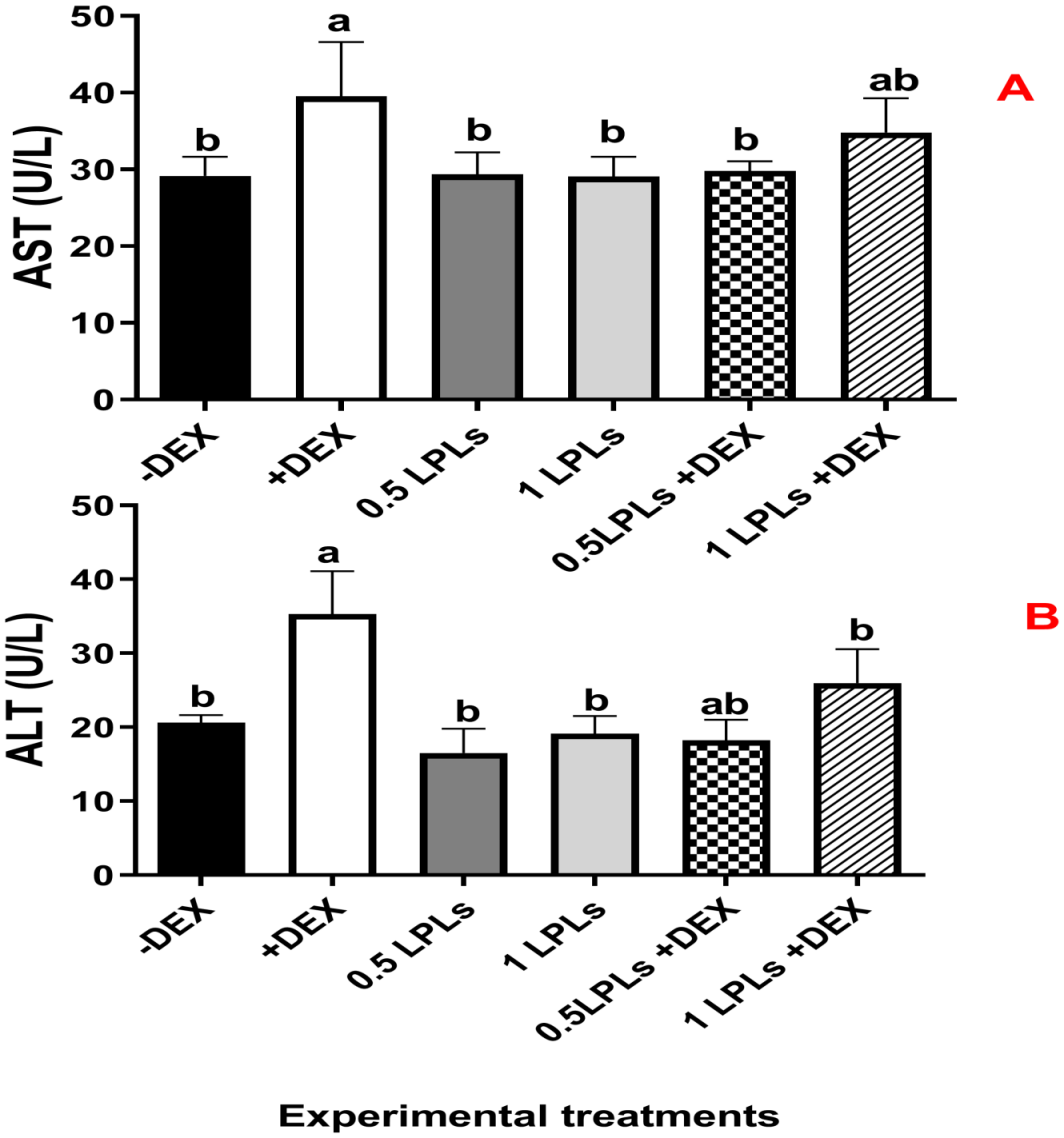
Effect of lysophospholipid dietary emusifiers on liver enzyme concentrations. -DEX denotes [negative control and fed basal diet (BD) only], +DEX (positive control, birds received BD containing 2 mg/kg dexamethasone); 0.5 LPLSs and 1 LPLs refer to birds fed BD containing 0.5 and 1 g of LPLSs/kg diet, respectively, whereas the DEX + 0.5LPLs and DEX + 1 LPLs represent birds receiving BD containing 2 mg/kg dexamethasone and supplemented with 0.5 and 1 g of LPLs/kg diet, respectively. The results are expressed as mean ± SE. Different letters denote statistical significance at *P <*0.05. *Post-hoc* power = 0.715 and 0.839 and partial eta squared (*η*²) = 0.481 and 0.646 for AST and ALT, respectively. Part **(A)** shows ALT cocentration (U/L) part **(B)** show AST concnetration (U/L).

The impacts of the separate and the concurrent dietary presence of LPLs, emulsifiers, and DEX on serum biochemistry are shown in **Table 3**. The birds received dexamethasone in their diet (positive control, +DEX) and exhibited significant increases in the levels of AST, ALT (**Figure 2**), creatinine, LDL-cholesterol, and triglycerides compared with their contemporaries that fed only on BD (negative control, -DEX) and those that received LPL emulsifiers with DEX in the diet at 0.5 and 1 g (*P* < 0.05). At the same time, the separate LPL dietary supplementation at 0.5 or 1 g did not alter the levels of these parameters compared to the control group (-DEX). On the other hand, the dietary presence of LPLs with DEX, particularly at 0.5-g supplementation level, significantly reversed the effects of dexamethasone by reducing the elevated levels of these parameters (*P* < 0.05). For total protein and albumen, there were no marked variations among the different groups, but the presence of DEX distinctly reduced the globulin levels, which were restored when LPLs were concurrently supplemented with DEX (*P* < 0.05).

**Table 3:** Effects of lysophospholipids (LPLs) on the serum biochemical parameters of broiler chickens subjected to oxidative stress.

Blood biochemical traits	Negative control (-DEX)	Positive control (+ DEX)	0.5 LPL_S_	1 LPL_S_	0.5 LPLs + DEX	1 LPLs + DEX	*p*-value	*Post-hoc* power	*η*²
Globulin (g/dL)	3.38 ± 0.09ab	2.58 ± 0.27c	3.58 ± 0.09a	3.40 ± 0.14ab	2.84 ± 0.18bc	2.81 ± 0.29bc	<0.01	0.959	0.732
Albumin (g/dL)	5.09 ± 0.08	4.43 ± 0.40	4.83 ± 0.41	4.08 ± 0.42	5.10 ± 0.36	4.30 ± 0.22	0.22	0.281	0.230
Total Protein (g/dL)	8.47 ± 0.40	7.76 ± 0.80	8.77 ± 0.27	7.73 ± 0.60	8.45 ± 0.45	7.76 ± 0.72	0.67	0.707	0.068
Creatinine (mg/dL)	0.58 ± 0.031b	0.94 ± 0.027a	0.56 ± 0.039b	0.56 ± 0.043b	0.65 ± 0.052b	0.63 ± 0.035b	<0.001	0.817	0.841
LDL- Cholesterol (mg/dL)	44.82 ± 2.37b	56.84 ± 4.90a	28.71 ± 1.15c	30.13 ± 3.38c	42.50 ± 5.05b	48.29 ± 3.46ab	<0.001	0.839	0.846
HDL Cholesterol (mg/dL)	40.00 ± 4.24ab	31.72 ± 4.568c	47.85 ± 3.12a	45.74 ± 2.51a	39.14 ± 3.82ab	29.42 ± 4.77b	<0.01	0.986	0.870
Cholesterol (mg/dL)	134.68 ± 4.17	144.23 ± 4.21	125.77 ± 9.16	126.82 ± 9.45	137.7 ± 7.38	131.75 ± 4.63	0.500	0.734	0.435
Triglyceride (mg/dL)	101 ± 1.87bc	116.5 ± 2.95a	99.0 ± 4.06c	103.75 ± 2.94bc	110.25 ± 2.71ab	116.5 ± 4.21a	<0.001	0.869	0.663

Values are expressed as means ± standard error. Mean values with different letters in the same row differ significantly at *P <*0.05.

DEX, dexamethasone; HDL, high-density lipoprotein; LDL, low-density lipoprotein; I/U, international units; *η*², partial eta squared.

### Phagocytic activity and index in response to dexamethasone and LPLs in diet

**Figure 3:**
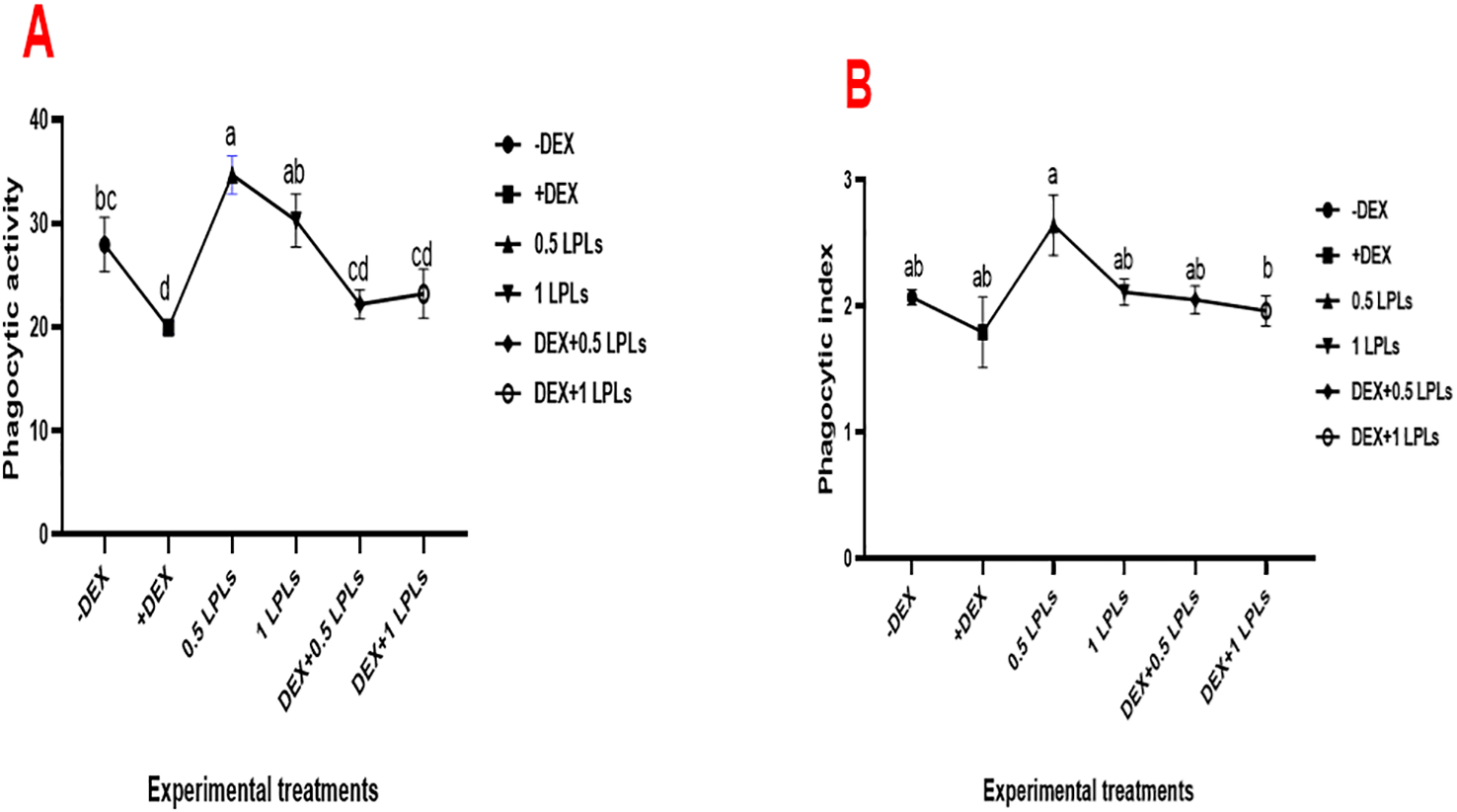
Effect of lysophospholipid dietary emusifiers on phagocytic activity and index. -DEX denotes [negative control and fed basal diet (BD) only], +DEX (positive control, birds received BD containing 2 mg/kg dexamethasone); 0.5 LPLs and 1 LPLs refer to birds fed BD containing 0.5 and 1 g of LPLs/kg diet, respectively, whereas the DEX + 0.5 LPLs and DEX + 1 LPLs represent birds receiving a BD containing 2 mg/kg dexamethasone and supplemented with 0.5 and 1 g of LPLs/kg diet, respectively. The results are expressed as mean ± SE. Different letters denote statistical significance at *P <*0.05. *Post-hoc* power = 0.939 and 0.853; partial eta squared (*η*²) = 0.713 and 0.485 for PA and PI, respectively. Part **(A)** show phagocytic activity. Part **(B)** shows phagocytic index.

**Figure 3** illustrates the effect of the separate and combined dietary supplementation of LPL emulsifiers with DEX on the phagocytic activity and index of broiler chicken. Supplementing the broilers’ diet with LPL emulsifiers, particularly at 0.5 g/kg diet, significantly increased PA and PI compared to the control (*P* < 0.05). However, the presence of DEX in the broilers’ diet induced marked reductions in the PA and PI (*P* < 0.05), which moderately recovered to normal with the combined supplementation of LPL emulsifiers with DEX in the diet (*P* < 0.05).

### Antioxidant enzyme activities and MDA concentration following the separate and concurrent supplementation of dexamethasone and LPL emulsifiers

**Figure 4:**
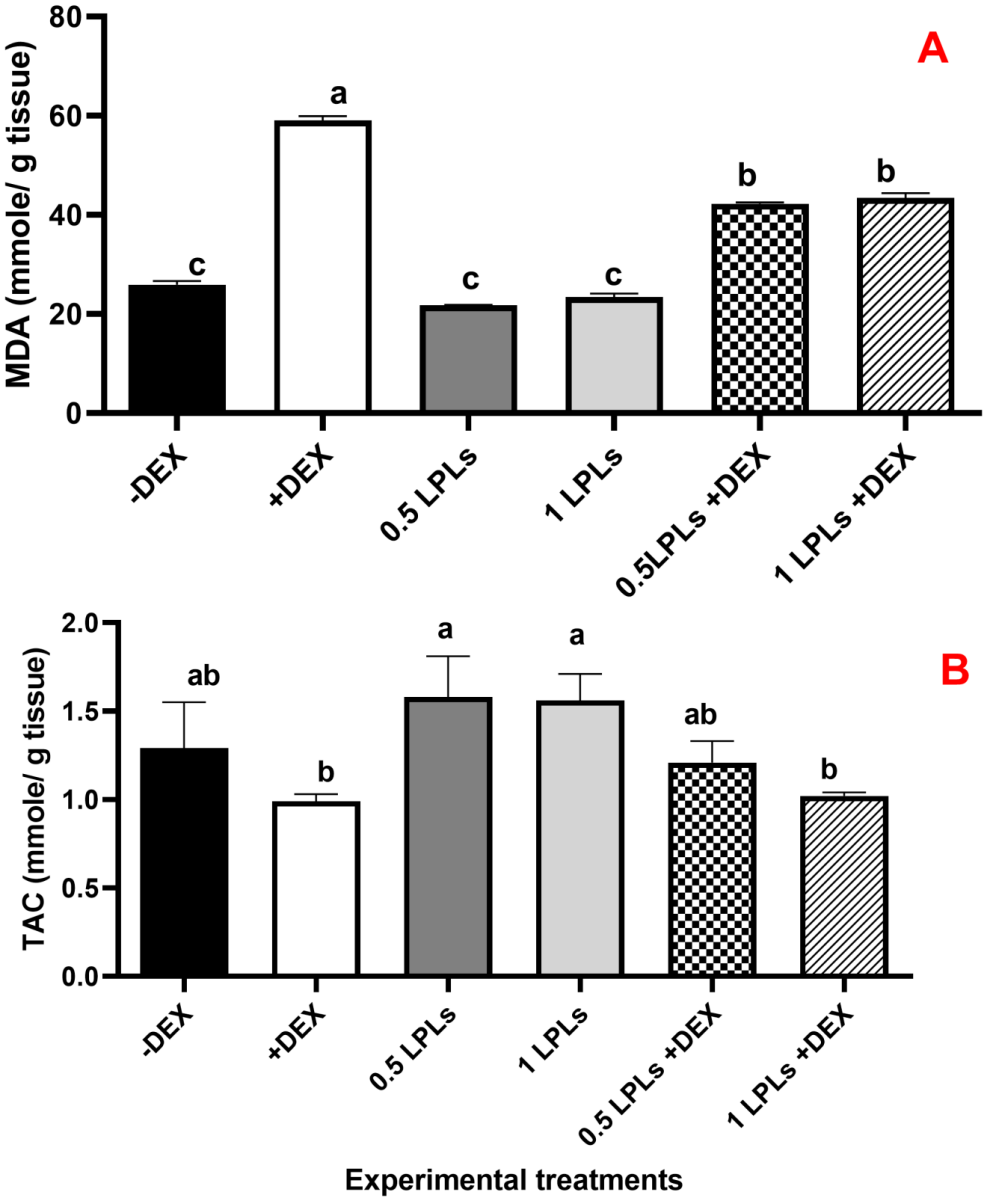
Effect of lysophospholipid dietary emusifiers on malondialdehyde and total antioxidant concentrations. -DEX denotes [negative control and fed basal diet (BD) only], +DEX (positive control, birds received BD containing 2 mg/kg dexamethasone); 0.5 LPLs and 1 LPLs refer to birds fed BD containing 0.5 and 1 g of LPLs/kg diet, respectively, whereas the DEX + 0.5 LPLs and DEX + 1 LPLs represent birds receiving a BD containing 2 mg/kg dexamethasone and supplemented with 0.5 and 1 g of LPLs/kg diet, respectively. The results are expressed as mean ± SE. Different letters denote statistical significance at *P <*0.05. *Post-hoc* power = 0.733 and 1.00; partial eta squared (*η*²) = 0.539 and 0.918 for TA and MDA, respectively. Part **(A)** show MDA concentration mmole/g tissue). Part **(B)** show total antioxidants (mmole/g tissue).

**Figure 5:**
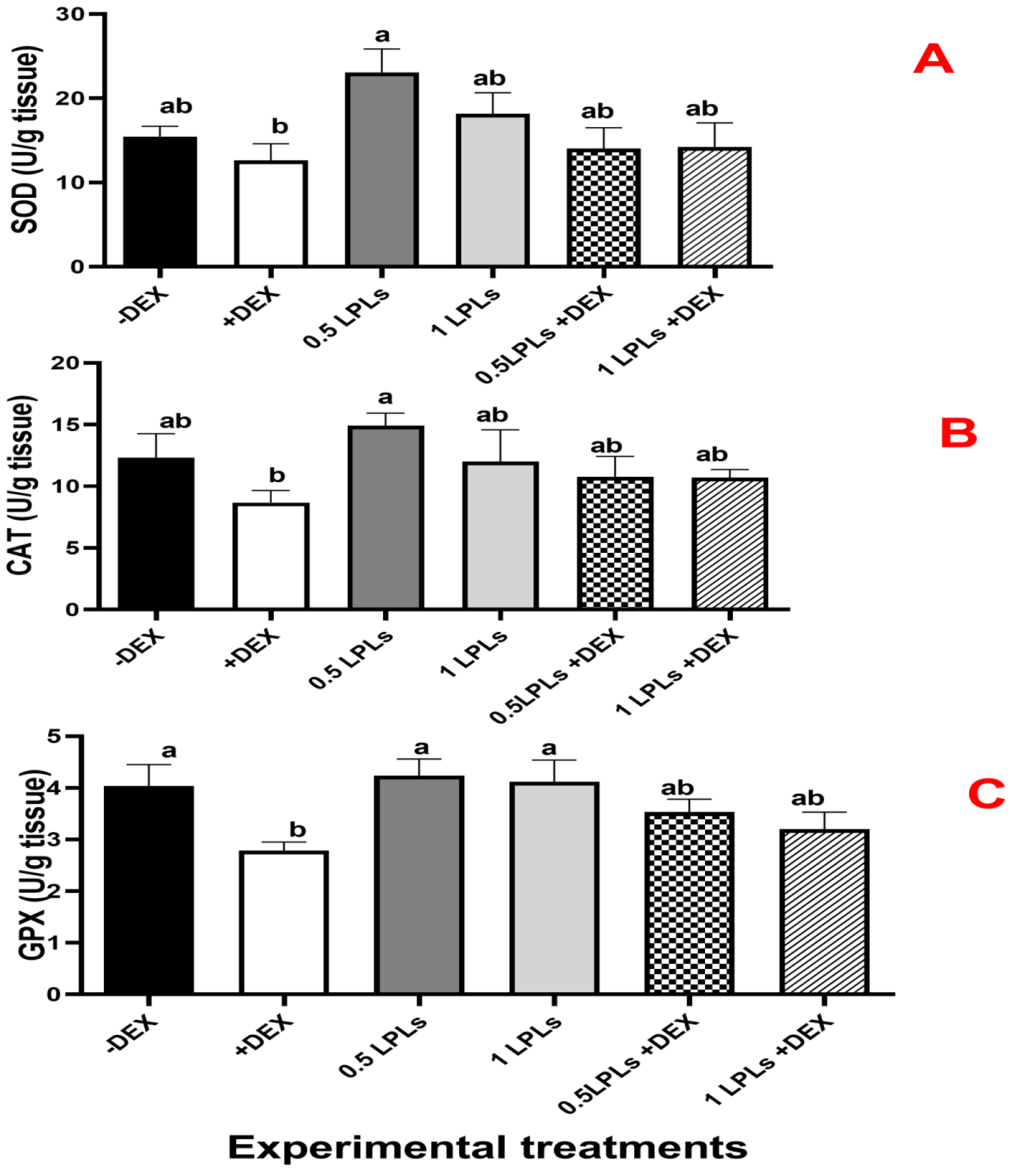
Effect of lysophospholipid dietary emusifiers on SOD, CAT, and GPx enzyme activity. -DEX denotes [negative control and fed basal diet (BD) only], +DEX (positive control, birds received BD containing 2 mg/kg dexamethasone); 0.5 LPLs and 1 LPLs refer to birds fed BD containing 0.5 and 1 g of LPLs/kg diet, respectively, whereas the DEX + 0.5 LPLs and DEX + 1 LPLs represent birds receiving BD containing 2 mg/kg dexamethasone and supplemented with 0.5 and 1 g of LPLs/kg diet, respectively. The results are expressed as mean ± SE. Different letters denote statistical significance at *P* < 0.05. *Post-hoc* power = 0.853, 0.849, and 0.803; partial eta squared (*η*²) = 0.492, 0.859, and 0.743 for GPX, CAT, and SOD, respectively. Part **(A–C)** show SOD, CAT, and GPX concentrations, respectively.

The effects of LPL emulsifier dietary supplementation on malondialdehyde (MDA) concentrations and total antioxidant (**Figure 4**) as well as antioxidant enzyme activities (**Figure 5**) in broiler chickens under DEX-induced oxidative stress were illustrated. The presence of dexamethasone in the diet noticeably induced oxidative stress as evidenced by a significant elevation in MDA concentration and clear reductions in TAC, SOD, CAT, and GPx activities (*P* < 0.05). An interesting return to normal status was reported with the combination of LPL emulsifiers with dexamethasone in the diet (at 0.5 and 1g/kg diet), confirmed by the non-significant elevations in the TAC, SOD, CAT, and GPx activities and reduction of MDA as compared to control (-DEX) and the two LPLs containing groups. However, it is clearly shown that the separate presence of LPL emulsifiers in broilers’ diets did not alter the TAC, SOD, CAT, and GPx activities, but resulted in a noticeable reduction in the MDA concentration compared to the other groups (P < 0.05).

### Histopathological features of intestine and liver tissues in response to dexamethasone and LPL emulsifiers

**Figure 6:**
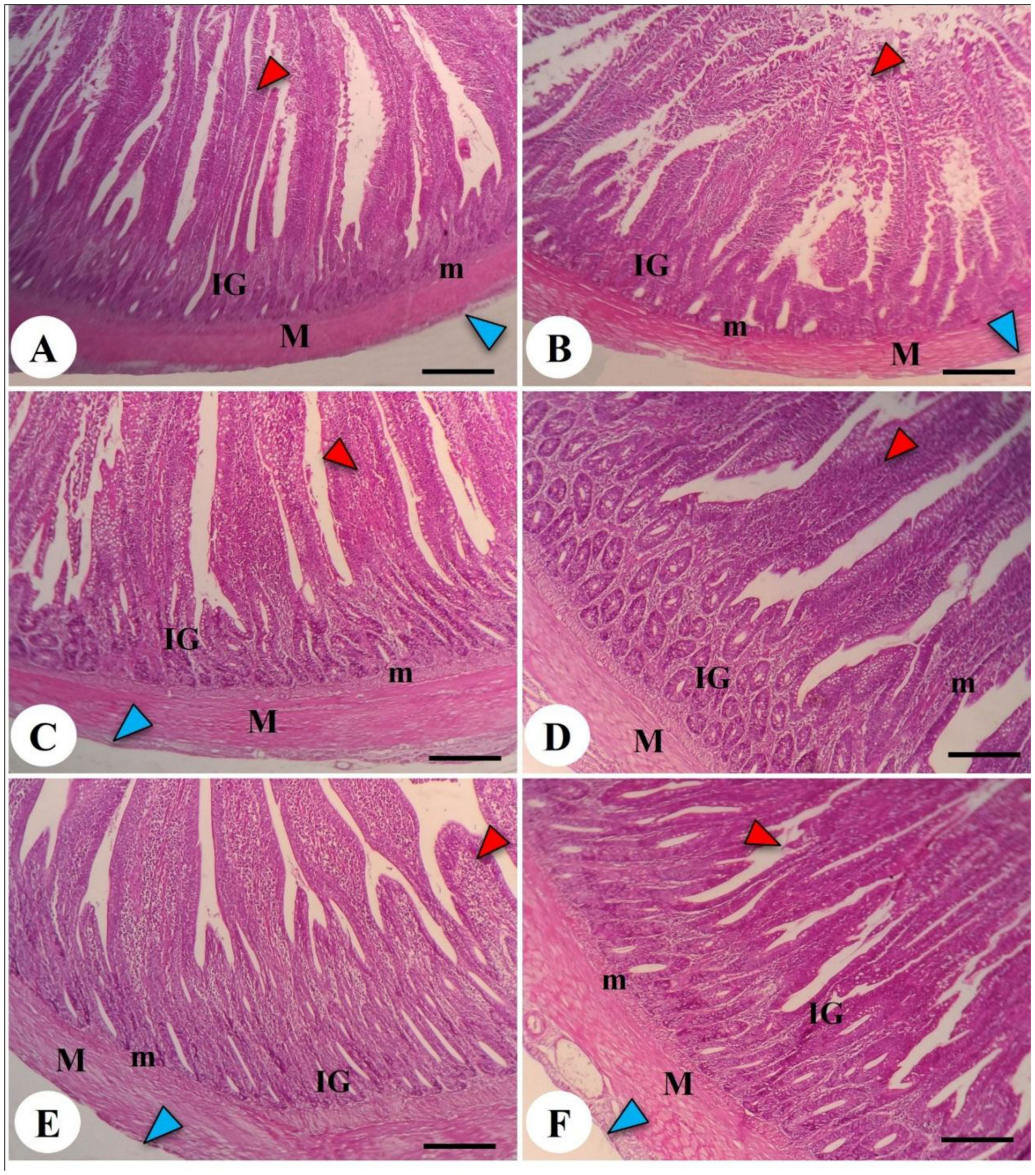
Photomicrograph of the intestine in the chicken broilers. The control negative (-DEX) **(A)**, control positive (+DEX) **(B)**, emulsifier-supplemented groups at 0.5 g LPLs/kg diet **(C)**, and high-level 1 g LPLs/kg diet **(D)** as well as groups subjected to 0.5 g LPLs + DEX **(E)** and 1 g LPLs + DEX **(F)** of emulsifier. The histological structure of the intestine presented the appearance of intestinal villi (red arrowhead), intestinal glands (IG), lamina muscularis mucosae (m), and muscularis externa (M), which was covered by serosa (blue arrowhead) outwardly. Stain, H&E. Bar, 100 µm.

**Figure 7:**
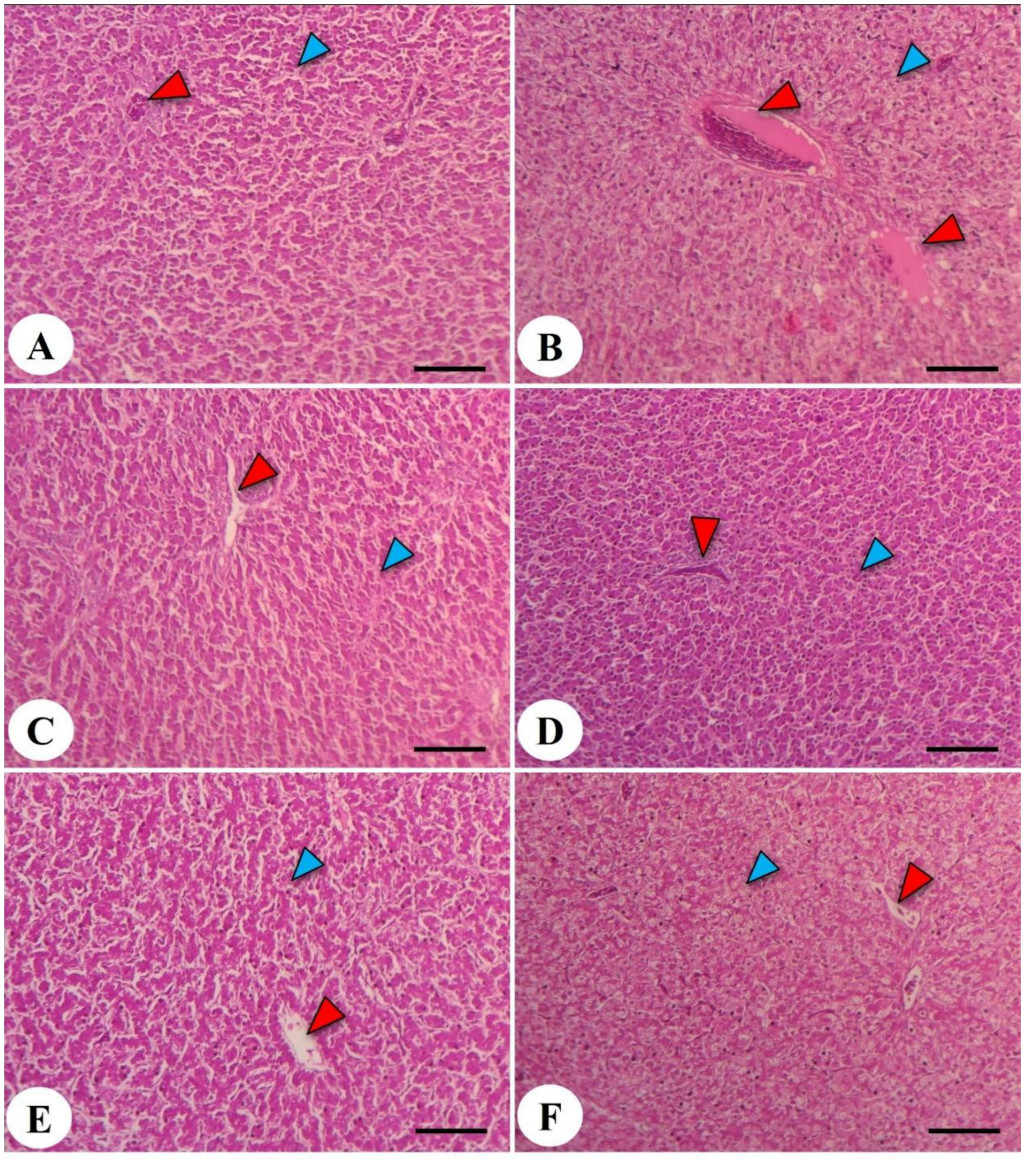
Hepatic photomicrograph of the liver in the chicken broiler. The control negative (-DEX) **(A)**, control positive (+DEX) **(B)**, emulsifier-supplemented groups at 0.5 g LPLs/kg diet **(C)**, and high-level 1 g LPLs/kg diet **(D)** as well as groups subjected to 0.5 g LPLs + DEX **(E)** and 1 g LPLs + DEX **(F)** of emulsifier. The histological structure of the liver showed the presence of hepatocytes (blue arrowhead) and a central vein (red arrowhead). Stain, H&E. Bar, 100 µm.

The effects of both LPL supplementation and DEX on the histopathological examination of jejunum and liver are shown in **Figures 6** and **7**, respectively. The histopathological investigation of the jejunum in the negative control (-DEX) group (**Figure 6A**) showed a normal structure of intestinal villi, which appeared as finger-like projections lined by simple columnar epithelium containing numerous goblet cells. The underlying propria submucosa contained simple tubular intestinal glands, with the lamina muscularis mucosae and tunica muscularis (muscularis externa) covered externally by serosa. However, the presence of DEX (+DEX) in the diet significantly deteriorated and disorganized the intestinal villi, with inflammatory cell infiltration in the lamina propria and degeneration of intestinal glands (**Figure 6B**). Whereas the separate LPLs-supplemented groups, particularly with 0.5g LPLs (**Figure 6C**), exhibited an improved morphology of the intestinal villi, with well-arranged enterocytes, enhancing the absorptive surface, and well-developed intestinal glands which occupied a larger area, particularly at 1g LPLs level (**Figure 6D**). An interesting alleviation of the DEX-induced tissue degeneration was noticed with the combined supplementation of LPL emulsifier with DEX, resulting in intact intestinal villi. In addition, it was observed that the lower level of LPL emulsifiers with DEX (0.5 g LPLs + DEX) partially restored the histological structure of the intestinal mucosa, with some inflammatory cell infiltration in the lamina propria (**Figure 6E**), whereas the higher level of LPL emulsifiers (1 g LPLs + DEX) significantly enhanced the histological architecture, with well-organized villi, glands, and goblet cells (**Figure 6F**). The morphometric analysis revealed augmented villous height, width, crypt depth, and muscular thickness of the intestinal wall in emulsifier-supplemented groups (**Table 4**). LPL separate supplementation (at 0.5-g level) induced significant increases in the villi length, base width of villi, villi crypt, and muscularis thickness compared to the 1-g supplementation level and the other groups (*P* < 0.05). However, the DEX-induced tissue degenerations were confirmed by the prominent reductions of villi length, base width of villi, villi crypt, and muscularis thickness compared to the other groups (*P* < 0.05), which were restored to normal in the case of the combined supplementation with LPL emulsifiers with DEX (*P* < 0.05).

**Table 4:** Effects of dietary supplementation with lysophospholipids (LPLs) on jejunal morphology in broiler chickens subjected to oxidative stress.

Parameter	Negative control (-DEX)	Positive control (+ DEX)	0.5 LPL_S_	1 LPL_S_	0.5 LPLs + DEX	1 LPLs + DEX	*p*-value	*Post-hoc* power	*η*²
Villi height (μm)	327.10 ± 8.65c	203.01 ± 6.58d	451.99 ± 5.66a	418.23 ± 9.15b	330.77 ± 3.61c	314.44 ± 8.21c	<0.001	1.00	0.984
Base width of villi (μm)	105.43 ± 2.96ab	52.61 ± 3.01d	115.61 ± 5.24a	100.34 ± 6.04b	103.44 ± 2.96ab	77.85 ± 3.17c	<0.001	1.00	0.930
Villi crypt (μm)	150.17 ± 3.75bc	60.56 ± 1.69e	160.52 ± 2.99a	157.75 ± 2.56ab	144.36 ± 3.77c	103.08 ± 1.65d	<0.001	1.00	0.988
Muscularis thickness (μm)	101.05 ± 5.58b	51.45 ± 2.07c	150.76 ± 0.64a	141.732 ± 5.24a	99.39 ± 5.69b	92.61 ± 5.75b	<0.001	1.00	0.962

Values are expressed as means ± standard error. Mean values with different letters in the same row differ significantly at *P <*0.05.

DEX, dexamethasone; *η*², partial eta squared.

The hepatic histopathological features of Avian 48 broilers fed the BD only without any additives (negative control, -DEX, **Figure 7A**) revealed normal hepatic parenchyma with polyhedral hepatocytes. These hepatocytes had centrally positioned nuclei and were organized into irregular, branching plates or cords, typically one or two cells thick, separated by blood sinusoids resembling capillaries around the central vein. However, the addition of dexamethasone at 2 mg/kg in the broilers’ diets (positive control, +DEX; **Figure 7B**) induced noticeable congestion and edema in the central veins, accompanied by degeneration, vacuolation of hepatocytes, and pyknotic nuclei. Notably, broilers supplemented with LPL emulsifiers in their diet at 0.5 and 1 g/kg diet showed hepatic structures similar to those fed the BD without any dietary supplementation (**Figures 7C, D**). Moreover, the dual presence of LPL emulsifiers in the broilers’ diet with DEX (**Figures 7E, F**) significantly alleviated the liver pathological degenerations caused by DEX, and the lower emulsifier level (0.5 g) proved a more beneficial mitigation than the 1-g level. The increased emulsifier levels (1 g) under the DEX-induced oxidative stress conditions induced mild fatty degeneration in hepatocytes.

### Impact of LPLs, emulsifiers, and dexamethasone on meat quality characterization

As presented in **Table 5**, the positive DEX group (+DEX) consistently showed the highest value for water-holding capacity, tenderness, pH, and color compared to all other treatments. When broiler chickens were supplemented with different concentrations of LPL emulsifiers and LPLs + DEX, the meat quality parameters showed lower values compared to the positive control, with measurements more closely aligning with the negative control group. Both LPL concentrations (0.5 and 1 g/kg diet) and their combinations with DEX showed similar trends.

**Table 5:** Effects of dietary supplementation of lysophospholipids (LPLs) on breast meat quality of broiler chickens subjected to oxidative stress.

Parameter	Negative control (-DEX)	Positive control (+ DEX)	0.5 LPLs	1 LPL_S_	0.5 LPLs + DEX	1 LPLs + DEX	*p*-value	*Post-hoc* power	*η*²
Water-holding capacity	11.12 ± 2.45b	17.20 ± 1.5a	8.50 ± 0.58b	8.50 ± 0.59b	8.3 ± 0.49b	9.36 ± 1.3b	<0.05	1.000	0.887
Tenderness	6.6 ± 2.00b	12.15 ± 1.75a	3.66 ± 1.02b	4.66 ± 0.751b	4.03 ± 1.05b	5.16 ± 1.31b	<0.05	0.999	0.833
PH	6.35 ± 0.15ab	6.60 ± 0.01a	6.20 ± 0.01b	6.26 ± 0.03b	6.43 ± 0.06ab	6.36 ± 0.03ab	<0.001	1.000	0.879
Color	0.396 ± 0.006b	0. 673 ± 0.127a	0.203 ± 0.028b	0.252 ± 0.089b	0.254 ± 0.038b	0.357 ± 0.003b	<0.05	1.000	0.864

Values are expressed as means ± standard error. Mean values with different letters in the same row differ significantly at *P <*0.05.

DEX, dexamethasone; *η*², partial eta squared.

## Discussion

Despite the wide expansion of broiler production to meet the continuous increase in human population, it is challenged by various kinds of stressors, which commonly result in oxidative stress (OS). OS is an indiscriminate biological reaction that results from the physiological imbalance between the activity of antioxidant enzymes and the production of reactive oxygen species (ROS) (49). High heat, humidity due to poor ventilation, and excessive use of glucocorticoids, such as dexamethasone (DEX), are common causes of OS in poultry, resulting in severe economic losses (50). These factors contribute to impairing the birds’ growth and immunity and increasing disease susceptibility and mortality, thereby reducing the meat quality by increasing lipid peroxidation (9). Dietary inclusion and supplementation of lipid emulsifiers have a promising role in alleviating the damaging effects of OS through increasing the activity of antioxidant enzymes and lipid digestibility as well as lowering ROS synthesis. Moreover, lipid emulsifiers enhance feed utilization, energy efficiency, and gut health, which collectively strengthen the birds’ defense against OS and improve their performance (27).

Accordingly, in this feeding study, the dietary presence of dexamethasone had an adverse effect on the birds’ growth parameters as evidenced by the reduced body weight and gain. The reduced body weight with the dietary presence of DEX might be correlated with reducing FI and impairing FCR (51). This DEX-associated reduction in FI might be linked with suppressing the hypothalamic orexigenic-regulatory neurons such as neuropeptide-Y (NPY) and Agouti-related peptide (AgRP) (52), which, in turn, might cause nutrient imbalance and insufficient nutrients for the higher growth rate of broilers (53). Therefore, future research is recommended to investigate the impact of dietary DEX presence on the expression levels and activities of hypothalamic orexigenic-regulatory neurons in depth. Moreover, the DEX-associated reduction in the broilers’ growth performance is possibly because of the degenerative changes in jejunum architecture as confirmed by the reduced villi length and width, crypt depth, and muscularis thickness (54). These changes also hinder the birds’ growth by reducing their intestinal absorptive capacity and nutrient availability (55). All of these factors suggest that DEX-associated OS compromises nutrient availability and energy efficiency and consequently retards growth performance. The reported impaired growth performance of broilers in response to feeding a diet containing DEX came in agreement with the findings of previous studies, which confirmed that dexamethasone induces oxygen species, which causes a reduction in the birds’ growth performance. Accordingly, the findings (56–58) confirmed the retarded growth in glucocorticoid-challenged broilers due to decreased feed intake and energy efficiency (59). Besides that, studies on Japanese quails and those conducted on broilers attributed the reduction in the birds’ growth performance following DEX exposure to increased protein catabolism, leading to muscular dystrophy and mobilization of fat stores. This explanation was confirmed by increasing the circulating protein catabolism marker, urate, due to the higher corticosteroid release associated with DEX-induced oxidative stress (60), leading to a reduction in muscle growth and body mass (61).

Under normal conditions, without DEX exposure, using dietary LPL emulsifiers, particularly at 1 g/kg diet, significantly improved the broilers’ growth performance as evidenced by the increased final weight, body gain, and improved FCR. The improvement in the birds’ growth in response to LPL emulsifiers may be due to increased nutrient digestibility, such as nitrogen and energy (62). The LPL-proposed increase in nutrient digestibility is perhaps correlated to enhancing the intestinal absorptive capacity as confirmed by the increase in villi length and width, crypt depth, and muscularis thickness in the LPL-treated groups, especially at the 0.5-g supplementation level of LPLs (63). The improved effect of LPLs on intestinal architecture may be attributed to the incorporation of LPLs into the cell membrane of enterocytes, altering the fluidity and permeability of the intestinal lipid bilayer and the protein channel functions (64), eventually increasing intestinal permeability and nutrient absorption, such as protein (65). Moreover, the reported improved effect of LPLs may be linked to its ability to promote the proliferation of intestinal epithelial cells, leading to increased intestinal mucosal height of broilers at 49 days old and preventing intestinal cellular damage (64, 66). These outcomes may indicate the potential improving influence of dietary emulsifiers, such as LPLs, on intestinal morphology, which, in turn, enhances growth performance by improving nutrient absorption, resulting in improved feed efficiency and body weight (63).

There are many studies that assessed the impact of dietary emulsifiers on birds’ FCR, growth, and nutrient digestibility (18, 20, 27, 67). Additionally, it has been reported that supplementing the single or blended emulsifiers with sodium stearoyl lactylate or glycerol monostearate improved the weight gain and FCR of broilers during the finisher and overall brooding period (68). Using emulsifiers such as lecithin and lysolecithin without antibiotic growth promoters also enhanced the broilers’ FCR and weight gain by increasing the ileal digestibility of nitrogen and energy (69). Furthermore, using emulsifiers such as bile acids with refined dietary oils enhances the birds’ growth performance and economic efficiency due to increased nutrient digestibility (70). The proposed association of increasing the intestinal morphometry indices and the reported improved growth with the dietary presence of LPLs also agreed with other literature reports, which reported increases in mucosal height and the height of jejunum villi, leading to enhanced intestinal digestion, absorption, and better utilization of nutrients (65, 67, 71, 72), such as energy (62) and protein (73), although other studies reported no changes in the birds’ weight gain and feed intake in response to the dietary supplementation of emulsifiers in a low-metabolizable energy diet (22, 74). This effect was observed in broilers up to 21 days of age (67, 75). The disparities between the findings of the various studies might be attributed to differences in the type of emulsifier utilized in each study (76), differences in the strain of broilers, and differences in the experimental design.

Interestingly, the combination of LPL emulsifiers with DEX in the broilers’ diet restored the impaired growth because of the DEX-associated OS by improving the DEX-linked damage in intestinal morphology, with consequent improvement of intestinal digestion, absorption, and nutrient utilization (63, 65, 67, 71, 72). The improving effect of LPLs on DEX-associated intestinal damage may be correlated with their ability to prevent intestinal cellular damage (64, 66). Additionally, the effect of LPLs in DEX-treated groups may be associated with their ability to improve intestinal tight junctions by increasing the expression of intestinal tight junction components, such as claudin-3, which is beneficial for paracellular transport and resistance to bacterial invasion (77). Increasing the expression of claudin-3 is necessary to ensure a tight seal of intestinal epithelial barrier and create a high concentration gradient of sodium ions across the epithelium that is required for the active transport of nutrients such as glucose and amino acids (65). Therefore, future studies are recommended to explore the synergistic protective mechanism of LPLs on DEX-associated oxidative stress by evaluating its effect on the expression of intestinal tight junction components, such as claudins, occludin, junctional adhesion molecule, and tricellulin as well as the cytoskeletal elements and cytoplasmic scaffolding proteins.

Carcass traits and meat quality are considered key economic factors for the broiler business (78). This current study clarified that the dietary presence of DEX significantly reduced the weights of the carcass, breast muscle, and thigh muscle, with marked increases in the weights of the liver and heart as well as the abdominal fat. The DEX-associated outcomes were reversed in the case of the separate and combined presence of LPL emulsifiers with DEX in the broilers’ diet. Reducing the carcass yield may be associated with oxidative stress resulting from feeding on a DEX-containing diet (58). These results were comparable to other literature reports, which indicated a retarded growth of thigh and breast muscle in response to feeding a DEX-containing diet due to reduced feed intake and feed efficiency (57, 79), whereas the reported enlargement of the liver and heart is probably correlated with increasing fat deposition in the liver as confirmed by increasing fatty changes in hepatocytes because of the DEX-linked oxidative damage. These findings are comparable with other observations reported in literature which stated that the increases in liver weight were due to the increases in liver lipids in stressed broilers (78, 80). Nonetheless, the dietary presence of LPL emulsifiers, either alone or with DEX, significantly increased and restored the carcass, breast, and thigh muscle weights and reduced both the hepatic and heart enlargement, along with lowering of abdominal fat. These effects may be correlated with facilitating the fractionation of lipids and protein and their conversion into the muscle tissue rather than abdominal fat deposition, which consequently affects the fatty acid and amino acid deposits in meat (72). This mechanism may also explain the protective effect of LPLs in preventing DEX-associated oxidative stress and hepatomegaly. Moreover, the reported improvement in carcass and relative organ weight following dietary supplementation with LPLs, with and without dexamethasone, is likely correlated with enhanced lipid emulsification and nutrient absorption as well as the increased bioavailability of energy substrates and the fat-soluble vitamins necessary for growth and tissue repair (65).

DEX also increased the water-holding capacity, tenderness, pH, and meat color. Increased pH is responsible for increasing the water-holding capacity and the color intensity (81, 82) due to splitting of meat proteins, causing a darker color (82). These outcomes align with those of Islam et al. (79), which indicated increases in color intensity and darker meat with the DEX treatment, whereas they disagree with the findings of Pan et al. (83), which reported increases in muscle lightness with DEX therapy. Additionally, increasing the water-holding capacity was the primary cause of the reported increase in meat tenderness (79). An interesting restoration of the meat quality indices, similar to those of the control group, was observed in the separate or combined LPL-treated broilers. The effect of LPL emulsifiers may be correlated with the even distribution of water and fat between muscle fibers, which reduces muscle catabolism and maintains a stable pH (84).

Serum biochemical parameters are a fundamental tool for reflecting oxidative stress due to alterations in blood biochemistry (78). Accordingly, in this study, DEX caused oxidative stress, resulting in lipid peroxidation and an increase in the levels of MDA, cholesterol-LDL, TG, and liver enzymes (ALT and AST). While there were no changes in the levels of total protein and albumen, there were reductions in the levels of globulin and HDL cholesterol. All of these findings were reversed and restored to normal (as in the control group, fed BD only) with the separate and combined supplementation of LPL emulsifiers with DEX. The elevated levels of the measured blood biochemical constituents in the case of DEX’s presence in the broilers’ diet might reflect the hepatic damage effects because of the ingestion of DEX (51), where the increases in the ALT and AST levels reflect the hepatic damage and oxidative stress in liver as confirmed by the reduced hepatic antioxidant enzyme activities, such as TAC, GPX, CAT, and SOD activities, with elevated MDA concentrations (85). Although the presence of LPL emulsifiers improved the serum biochemical profiles in broilers under dexamethasone (DEX)-induced oxidative stress by reducing the LDL-cholesterol, triglyceride, and liver enzyme levels while enhancing HDL-cholesterol. In this context, the 0.5 g LPLs + DEX showed the most consistent benefits by effectively attenuating the oxidative stress effects. These findings underscore the protective potential of LPL emulsifiers against oxidative damage by reducing the blood LDL levels (23). Similarly, feeding broilers a diet containing 2% lecithin significantly reduces their blood cholesterol levels (26). Another study found that 0.5% lecithin significantly increased the glucose, total cholesterol, triglycerides, HDL, LDL, and VLDL levels by 4%, 9%, 7%, 24%, 25%, and 29% respectively (86). Additionally, dietary supplementation with lysophospholipids has been shown to improve the serum glucose levels and high-density lipoprotein (HDL) concentrations in broilers (72). However, other studies reported that lysolecithin inclusion did not affect the serum total cholesterol and triglyceride levels (87). Additionally, other studies have indicated that there were no alterations in the levels of cholesterol, glucose, and triglycerides in broilers fed diets containing emulsifiers or emulsifiers with lipase (88). These diverse findings are likely correlated with the complex metabolic effects of emulsifiers, which vary based on the different doses of emulsifiers, the route of supplementation, and individual physiological variations.

Regarding the activities of antioxidant enzymes and lipid peroxidation in liver tissue, LPL dietary supplementation at 0.5 and 1 g/kg diet markedly enhanced the antioxidant enzyme activities and reduced the MDA levels, thereby counteracting the effects of DEX in broilers. These effects perhaps conclude with the LPL-improving effect through inducing oxidative balance as confirmed by the higher TAC, GPX, CAT, and SOD activities and the decreased MDA levels (89). Various studies have demonstrated the modulatory effect of dietary emulsifiers on oxidative stress in broilers by enhancing the activities of antioxidant enzymes—for example, Ewais et al. (90) documented that a limonene nano-emulsion significantly increased the levels of various antioxidant enzymes, such as GPX, CAT, and SOD, with a further reduction in MDA levels. Moreover, the dietary supplementation of lysolecithin at 300–400 g/100 kg beneficially lowered the MDA and increased the SOD and GPX activities (91). The de-oiled lecithin dietary supplementation also differentially increased the SOD, GPx, and TAC activities with obvious reduction in the MDA concentration among two different broiler strains (92). The reported antioxidant efficacy of emulsifiers might be attributed to their unique molecular composition, for example, lecithin contains gamma, alpha, and delta tocopherols; it derives its primary antioxidant function from the synergistic action of gamma and delta tocopherols combined with amino-alcohol phospholipids (92). The presence of amino groups and choline in phospholipids enables them to function as effective lipophilic antioxidants, defending against oxidative stress and free radical damage. Additionally, lysolecithin’s antioxidant benefits are ascribed to its ability to reduce liver damage, enhance oxidative resistance, and improve the oxidative stability of oils and fats through the protective action of its phospholipid constituents (93). Additionally, LPLs’ antioxidant properties may be linked to its ability to bind to pro-oxidative metals through the negative charges found on its phosphate head group, resulting in the inhibition of lipid oxidation and reduction in electron leakage and reactive oxygen species (ROS) production by improving mitochondrial β-oxidation efficiency (94). LPLs can also indirectly combat oxidative stress through their anti-inflammatory characteristics as inflammation can induce oxidative stress through the activation of several pathways, such as the transcription factor NF-κB, which activates the reactive species (89). LPLs such as phosphatidylcholine inhibit oxidative stress via inhibiting the TNF-α-induced proinflammatory signaling and lowering the activities of specific pro-inflammatory components such as IL-8, ICAM-1, IP-10, MCP-1, and TNF-α (89). Accordingly, Boontiam et al. (72) found that dietary supplementation of lysophospholipids in a low-energy and nitrogenous diet enhances the antioxidant response by alleviating inflammation through the reduction of interleukin-1 levels. Furthermore, the reported increases in the activities of SOD, CAT, and GPx in the LPL-supplemented groups (separately and combined with DEX) might be attributed to its ability to activate the Nrf2–Keap1 signaling pathway (95).

At the level of the non-specific immune response, our results showed that the dietary supplementation with LPL emulsifiers improved the phagocytic index (PI) and activity (PA) in broilers under DEX-induced oxidative stress. This result proved that LPLs counteracted the DEX-associated immunosuppressive effects. Accordingly, both 0.5 and 1 g LPLs/kg diets partly restored the PA and PI in the DEX-exposed broilers, with the 0.5 g LPLs + DEX treatment enhancing PI more effectively, while the 1 g LPLs + DEX treatment improved the PA. The modulatory effect of LPLs, either alone or when combined with DEX-linked OS, might be correlated with its several mechanisms. Accordingly, LPLs, due to their amphiphilic characteristics, can be integrated into phagocyte cell membranes, thereby enhancing their fluidity and structural integrity and promoting phagocytosis and phagosome formation (65). Additionally, due to its antioxidant properties, LPLs can attenuate oxidative stress within phagocytes by maintaining the intracellular redox balance (92), thereby facilitating the formation of an oxidative burst that is necessary for the phagocytic process and microbial killing (31). Besides that, LPLs may modify the non-specific immune response, including PA and PI, by modulating the signaling pathways responsible for cytokine production, such as NF-κB and MAPK, which enhance the innate immune response and suppress the dexamethasone-associated oxidative stress (72, 89). Lastly, LPLs could enhance phagocytosis by improving nutrient absorption, thereby ensuring the availability of essential nutrients, such as protein and energy, that are necessary for phagocytosis. Collectively, these LPLs’ characteristics and influences could explain their immunomodulatory role in preserving the innate immune response, particularly under oxidative stress in broilers, by promoting the birds’ immunity and health (96).

## Conclusions

From the foregoing results, it can be concluded that the dietary incorporation of lysophospholipid emulsifiers (LPLs), particularly at 0.5 g/kg diet, can effectively alleviate dexamethasone-induced oxidative stress and improve the birds’ performance. These effects were evidenced by restoring the dexamethasone-retarded growth performance, improving the feed conversion efficiency, and enhancing the intestinal absorptive capacity through improved intestinal morphology. LPL emulsifiers (particularly at 0.5 g/kg diet) also enhanced the antioxidant capacity by increasing the antioxidant enzyme activities of SOD, CAT, and GPX and the phagocytic activity, which mitigated the DEX-induced oxidative stress through improving the bird’s redox system to scavenge the excessive synthesis of ROS and preventing lipid peroxidation as confirmed by reducing the DEX-elevated MDA concentration. Additionally, LPL emulsifiers enhanced the bird’s carcass performance and meat quality by maintaining the meat’s water-holding capacity, pH, and tenderness. Thus, the dietary incorporation of LPL emulsifiers, particularly at a concentration of 0.5 g/kg in the diet, could serve as an effective and practical solution to alleviate oxidative challenges, improve the production efficiency, and enhance the excellence of end-products, thereby supporting global food security and nutritional well-being.

## Data Availability

The raw data supporting the conclusions of this article will be made available by the authors, without undue reservation.
